# Beyond individual correlates: The moderating roles of neighborhood factors on loneliness among adults in predominately Black neighborhoods in Pittsburgh

**DOI:** 10.1016/j.healthplace.2026.103646

**Published:** 2026-03-25

**Authors:** Moka Yoo-Jeong, Greta Jianjia Cheng, Tamara Dubowitz, Wendy M. Troxel, Gerald Hunter, Bonnie Ghosh Dastidar, Tanisha G. Hill-Jarrett, Andrea L. Rosso

**Affiliations:** a School of Nursing, Bouvé College of Health Sciences, Northeastern University, Boston, MA, USA; b Social Environment and Health Program, Survey Research Center, Institute for Social Research, University of Michigan, Ann Arbor, MI, USA; c Department of Epidemiology, School of Public Health, University of Pittsburgh, Pittsburgh, PA, USA; d RAND Corporation, Health Division, Pittsburgh, PA, USA; e RAND Corporation, Pittsburgh, PA, USA; f Memory and Aging Center, Department of Neurology, University of California San Francisco, USA; g Global Brain Health Institute, University of California San Francisco, USA

**Keywords:** Loneliness, Physical function, Neighborhood perceptions, Socioeconomic deprivation, Residential stability

## Abstract

While individual correlates of loneliness are well established, less is known about whether neighborhood characteristics modify these associations. Guided by the Ecological Theory of Aging, this study examined whether subjective and objective neighborhood factors moderated cross-sectional associations between key individual characteristics (age, gender, physical function) and loneliness among adults in two predominantly Black Pittsburgh, PA neighborhoods. Data were drawn from the Think PHRESH study (N = 694), an ancillary to a longitudinal cohort study of the Pittsburgh Hill/Homewood Research on Neighborhood Change and Health (PHRESH). Loneliness was measured using the UCLA 3-item scale. Subjective neighborhood factors included perceived safety and neighborhood satisfaction. Objective factors included neighborhood socioeconomic deprivation and residential instability from Census data. Multivariable linear regressions with interaction terms tested moderation effects, followed by stratified analyses for significant interactions. Among those perceiving their neighborhoods as less safe, middle-aged adults (50–65 years) and older adults (≥65 years) reported significantly higher loneliness compared to younger adults (31–49 years). These age-related differences were not observed among those reporting high safety. A similar pattern emerged for neighborhood satisfaction: greater loneliness was reported among middle-aged and older adults with low neighborhood satisfaction, but not among those with high satisfaction. Physical limitations were also linked to greater loneliness among those with low perceived safety or neighborhood satisfaction. Women living in more deprived neighborhoods reported higher loneliness than men, while no gender differences were observed in less deprived areas. These findings suggest that neighborhood perceptions may be salient in shaping loneliness and that neighborhood disadvantage may exacerbate gendered vulnerabilities to loneliness. Future research should investigate the mechanisms through which neighborhood perceptions develop and influence loneliness over time, and evaluate interventions addressing both individual and contextual determinants of loneliness in historically marginalized communities.

## 1. Introduction

Loneliness, defined as the negative experience due to perceived lack of desired social connection (Cacioppo et al., 2014; Perlman and Peplau, 1981), is increasingly recognized as a significant public health concern with implications for both mental and physical health (Cacioppo et al., 2006; Hawkley and Cacioppo, 2010; Holt-Lunstad, 2024; The, 2023). Loneliness prevalence varies across age, gender, and physical function (Hawkley et al., 2022; Luhmann and Hawkley, 2016; Pinquart and Sörensen, 2003; Qualter et al., 2015). For example, national surveys suggest a U-shaped trajectory across the lifespan, with younger and oldest-old adults reporting higher loneliness than those in midlife (Graham et al., 2024; Luhmann and Hawkley, 2016). Rather than representing inherent vulnerability, these characteristics intersect with broader environmental factors, such as neighborhood, that can amplify or mitigate loneliness (Atshan et al., 2025; Feng and Astell-Burt, 2022).

The Person-Environment Fit, or the Ecological Theory of Aging (ETA) (Lawton and Nahemow, 1973), provides a useful framework for understanding how neighborhood environments may moderate the effects of individual factors on loneliness, either exacerbating or buffering the risk for loneliness (Lawton and Nahemow, 1973). ETA posits that an individual’s well-being and health as one grows older are influenced by the interplay and dynamic interaction between personal competencies and environmental press—the demands or supports offered by the surrounding environment (Wahl et al., 2012). When environmental press exceeds an individual’s capabilities—for example, perceived unsafe neighborhoods that may further restrict mobility in older adults— his or her well-being declines. Conversely, when the environment provides social and structural supports that align with individual needs, it can enhance well-being and buffer against negative outcomes (Wahl et al., 2012).

In the context of loneliness, the ETA suggests that neighborhood characteristics may shape how individual attributes translate into psychosocial outcomes. Rather than viewing these attributes as inherent “risk factors,” this framework positions neighborhood conditions as contextual moderators that can either amplify or buffer against loneliness. For example, supportive neighborhoods characterized by high perceived safety and satisfaction can mitigate loneliness by facilitating social engagement, fostering belonging, and promoting informal social interactions (Meehan et al., 2023). Similarly, inclusive and accessible built environments can protect against loneliness by providing opportunities for diverse social encounters (Kearns et al., 2015). However, the lack of such resources—as seen in economically deprived neighborhoods—may exacerbate loneliness by constraining social participation and reinforcing isolation (Victor and Pikhartova, 2020). High residential turnover can further weaken place-based ties and diminish a sense of belonging (Beer et al., 2011; Kang, 2022). Therefore, it is essential to examine both subjective and objective neighborhood characteristics not solely as direct determinants of loneliness but also as contexts in which other factors operate to influence loneliness.

Subjective neighborhood characteristics refer to individual’s perceptions of their immediate environment, including feelings of safety, satisfaction, and social cohesion. These perceptual factors capture how individuals interpret and experience their surroundings in ways that affect their sense of connection and belonging. Prior research indicates that lower perceived neighborhood safety and satisfaction are associated with higher loneliness, likely through reduced trust, fear of victimization, and limited social engagement opportunities (Meehan et al., 2023). In contrast, objective neighborhood characteristics describe structural and compositional features of the built and social environment, such as socioeconomic deprivation, residential instability, and access to community resources. These indicators, derived from census and administrative data, capture broader material conditions that shape the social infrastructure of communities. Disadvantaged or unstable neighborhoods may constrain opportunities for interaction and exacerbate social isolation, whereas resource-rich, stable neighborhoods may foster connection and mutual support (Kearns et al., 2015; Victor and Pikhartova, 2020). Although subjective and objective measures are conceptually distinct, they are interrelated: perceptions of neighborhood quality often mirror, but do not fully correspond to, structural conditions. Examining both provides a more comprehensive understanding of how neighborhood context contributes to loneliness through social and environmental pathways.

Neighborhood conditions may amplify or buffer against the feelings of loneliness in certain populations. Older adults often experience increased dependence on their immediate environment due to diminishing social networks and declining mobility, making neighborhood characteristics a critical determinant of their social well-being (Cornwell and Waite, 2009). Evidence regarding gender and loneliness remains mixed. Meta-analytic findings indicate that mean differences in loneliness between men and women are generally small and context dependent, shaped by social and cultural expectations (Maes et al., 2019). Gender reflects socially patterned opportunities for connection and engagement that may intersect with neighborhood context (Pinquart and Sörensen, 2003). For example, women’s social participation may often involve caregiving networks, informal neighborhood support, and community involvement—domains that rely on neighborhood safety, cohesion, and stability. In contrast, men’s social ties may be more affected by role or work-related transitions. Thus, neighborhood environments may influence loneliness differently across gendered social pathways, not because one group is inherently more vulnerable, but because environmental conditions shape how social relationships are maintained. Similarly, individuals with physical limitations may be particularly dependent on the accessibility, safety, and stability of their neighborhood for maintaining social connections (Cloutier-Fisher et al., 2011).

Understanding the role of these neighborhood environment factors is particularly important in historically marginalized communities, where structural inequalities may exacerbate loneliness. As such, the present study aimed to explore the influence of subjective versus objective neighborhood factors on loneliness, and examine whether these neighborhood contexts moderate associations between key individual characteristics (age, gender, and physical limitations) and loneliness in adults (≥35 years old) living in two predominately Black neighborhoods in Pittsburgh. We hypothesized that subjective (e.g., neighborhood satisfaction and safety) and objective (e.g., neighborhood socioeconomic deprivation and residential instability) neighborhood factors will moderate the relationship between individual factors and loneliness.

## 2. Methods

### 2.1. Data sources

The data utilized in the current study originate from Think PHRESH (Rosso et al., 2023), an extension of a longitudinal cohort study initiated in 2011 called the Pittsburgh Hill/Homewood Research on Neighborhood Change and Health (PHRESH) project (Dubowitz et al., 2015). PHRESH tracks a geographically representative, probability-based sample of residents from two predominantly Black, low-income neighborhoods in Pittsburgh—the Hill District and Homewood. These neighborhoods were originally chosen due to their comparable geographic locations and sociodemographic characteristics. Since the study inception, the Hill District has experienced significant investments, including the establishment of a supermarket, housing redevelopment, enhancements to green spaces, and commercial revitalization. Similarly, Homewood has undergone various neighborhood investments which have occurred largely between 2016 and 2022. Data collection has been conducted every one to two years and remains ongoing, encompassing comprehensive evaluations of both individual and neighborhood characteristics across multiple study waves, as well as assessments of health behaviors and outcomes at specific time points.

Think PHRESH, started in 2022, collecting data via surveys and cognitive assessments from active PHRESH participants aged 35 and older, and a sample of participants newly recruited in 2024. For the current analysis, survey data from 814 participants who completed the Wave 1 of Think PHRESH were utilized. The final analytic sample included 694 participants who had complete data on neighborhood subjective measures, addresses that were able to be geocoded for objective neighborhood measures, and covariates. The study protocol was approved by the Institutional Review Boards of both RAND and the University of Pittsburgh. All participants provided informed consent prior to participation. Trained data collectors, who were either residents of or familiar with the neighborhood, conducted all surveys either in participants’ homes or at nearby community centers. This approach facilitated rapport and ensured accurate data collection.

### 2.2. Measures

#### 2.2.1. Loneliness

Loneliness was assessed using the University of California, Los Angeles (UCLA)-3 item Loneliness Scale, which is an adaptation of the 20-item Revised UCLA Loneliness Scale (Russell et al., 1980), that shows strong psychometric properties (Hughes et al., 2004). Responses were rated on a 3-point scale from 1 (Hardly ever), 2 (Some of the time), and 3 (Often) (Cronbach’s alpha: 0.77). Scores were summed across the three items, yielding a total score ranging from 3 to 9, with higher scores indicating greater loneliness.

#### 2.2.2. Person-level factors

Age in years was categorized into three age groups (age 31–49, age 50–65, and age ≥65). Gender was self-reported as man or woman. Self-reported physical function was measured by a 3-item self-reported questionnaire, which was adopted from the SF-36 Health Survey (Syddall et al., 2009; Ware and Gandek, 1998). In this questionnaire, participants were asked, “does your health limit you a lot, a little, or not at all when doing moderate activities (e.g., moving a table, dancing), climbing several flights, and walking several blocks?” Based on their response, participants were grouped into the following categories: “no limitations” if they reported being “not limited at all” across three types of activities; “moderate limitations” if they reported “limited a little” in any of the activities; and “severe limitations” if they reported “limited a lot” across all three activities.

#### 2.2.3. Subjective neighborhood factors

*Neighborhood satisfaction* was evaluated using an item from the Los Angeles Family and Neighborhood Survey (LAFANS) questionnaire (Sastry et al., 2006). This item measured participants’ overall satisfaction with their neighborhood by asking participants, “All things considered, would you say you are very satisfied, satisfied, dissatisfied, very dissatisfied, or neutral with your neighborhood as a place to live?” Responses were recorded on a 5-point scale, ranging from 1 (Very dissatisfied) to 5 (Very satisfied), with higher score indicating a greater neighborhood satisfaction. Due to small sample sizes in some of the categories, neighborhood satisfaction was dichotomized as dissatisfied/very dissatisfied/neutral versus satisfied/very satisfied in the analyses to have adequate cell sizes.

*Perceived neighborhood safety* was assessed using four items from the Perceived Neighborhood Scale (PNS) (Martinez et al., 2002). These items measure individuals’ perception of safety within their neighborhood by asking about their perceived safety in walking during the day and evening, safety from crime and violence. Responses were recorded on a 5-point Likert scale, ranging from 1 (Strongly disagree) to 5 (Strongly agree) (Cronbach’s alpha: 0.71). Scores were calculated by averaging responses across all four items, with higher scores indicating a greater perceived level of neighborhood safety. We dichotomized the continuous score using the median value as the cut-off point and included the dichotomized score (below median vs. above median) in the analyses, with above median reflecting better safety.

#### 2.2.4. Objective neighborhood factors

The socioeconomic deprivation and residential instability indexes represent a combination of midlife and late-life neighborhood exposures according to participant’s age. Address information was collected from a self-reported residential history questionnaire and linked to US census data by tract. Each address was matched to the closest available census year and was aligned by year with participant’s specific life course periods including middle adulthood (ages 30–55) and older adulthood (ages 56+). Specifically, addresses from age 30 through the current age were geocoded and linked to the closest census year to create the indexes for participants aged 55 or younger. For participants aged 56 years and older, addresses from age 56 onward were geocoded and linked to the closest census year to create the indexes. Neighborhood indices therefore reflect cumulative exposure across adulthood or later adulthood, rather than point-in-time conditions.

*Neighborhood socioeconomic deprivation* was calculated as the average score of 14 standardized census measures that were preselected in a principal component analysis by Singh (2003). All measures were z-score standardized to the national level. These 14 census measures can be viewed as approximating the material and social conditions and relative socioeconomic disadvantage in each census tract. A higher score indicates higher socioeconomic deprivation. We dichotomized the continuous score using the median value of the sample as the cut-off point and included the dichotomized score (below median vs. above median) in the analyses, with above median reflecting more socioeconomic deprivation.

*Neighborhood residential instability* was calculated as the average score of five standardized census measures that represent mobility, housing tenure, and occupancy in a given census tract. A higher score indicates higher residential instability. Measures include percent of all units which are vacant, percent of renter-occupied units, percent of population who moved from a different county in previous year, percent of population who moved in previous year, and percent of population who moved within last 5 years. All measures were z-score standardized to the national level. We dichotomized the continuous score using the median value as the cut-off point and included the dichotomized score (i.e., below median vs. above median) in the analyses, with above median reflecting greater instability.

#### 2.2.5. Covariates and confounders

Covariates were selected to account for potential confounding in fluences on the association between neighborhood characteristics, individual factors, and loneliness. These included demographic and socioeconomic indicators (race, marital status, education level, household income, neighborhood of residence), social support, and psychological distress. Race was controlled for to account for within-sample heterogeneity and differences in neighborhood racial composition. Social support was included to account for perceived interpersonal resources. Psychological distress was included to adjust for general negative affective that could influence loneliness or neighborhood perceptions. Household income was modeled in thousands of dollars. Education level was categorized as less than high school, high school graduate, 1–3 years of college, and college degree or greater. Marital status was categorized as married or living with a partner, windowed, divorced or separated, and never married. Neighborhood was included as a design variable and included Hill District, Homewood, and other; these neighborhoods are geographically larger than the census tracts at which the objective neighborhood variables are calculated.

*Social* support was assessed using the four item Tangible Support Subscale from Interpersonal Support Evaluation List (ISEL) (Cohen et al., 1985). ISEL is a measure of perceptions of social support and contains three different subscales measuring three aspects of perceived social support, including Appraisal Support, Belonging Support, and Tangible Support Subscales. In PHRESH, only Tangible Support Subscale was administered. This subscale includes questions asking participants whether there is someone available to support when needing help in situations such as being sick, out of town, stranded, and moving. Responses were rated on 4-point scale ranging from 1 (Definitely false) to 4 (Definitely true). These four items were summed, with higher scores reflecting greater support.

*General psychological distress* was assessed using the K6 Psychological Distress Scale, a six-item tool developed to assess nonspecific psychological distress, which was originally developed for the US National Health Interview Survey (NHIS) (Kessler et al., 2002). This scale includes questions asking participants how often they experienced feelings such as being “nervous,” “hopeless,” or “so depressed that nothing could cheer you up” in the past 30 days. Responses were recorded on a 5-point scale, ranging from 1 (None of the time) to 5 (All of the time). The total score was obtained by summing responses across all six items, resulting in a possible range of 0 to 24, with higher scores reflecting greater psychological distress.

### 2.3. Data analysis

Descriptive statistics were conducted to characterize the full sample with perceived neighborhood measures and in the subsample with neighborhood objective measures. First, unadjusted (bivariate) regression models were estimated to examine the crude associations between loneliness and each predictor variable (age, gender, physical limitations, subjective and objective neighborhood characteristics). Second, adjusted (multivariable) models were estimated that included all predictors simultaneously and controlled for covariates (age, education, employment, income, race, social support, and psychological distress). As objective neighborhood data represented a lifecourse period and may have been derived from multiple census tracts, we did not use multilevel modeling. Instead, neighborhood was modeled as a fixed effect, and objective neighborhood characteristics were treated as continuous census-tract–level variables assigned to each participant. Moderation analyses tested whether subjective and objective neighborhood factors modified the associations between individual-level characteristics (age, gender, physical limitations) and loneliness by including the multiplicative interaction term between each individual-level factor and each neighborhood-level factors in separate regression models. When effect modification was indicated by a significant interaction term, we ran stratified analyses on those neighborhood factors to demonstrate the effect modification. All neighborhood factors were included as dichotomous variables to facilitate stratification and interpretation of effect modification. All analyses were performed using Stata SE 18.0, and statistical significance was set at *p* < 0.05.

## 3. Results

Table 1 presents sample characteristics for the Think PHRESH participants overall (n = 814) and for our analytic sample (n = 694). The average age of our analytic sample was 63 years (SD:13) and 95% self-reported as Black/African American race. Approximately half of the sample was aged 65 and older, and 76% identified as a woman. The mean loneliness score was 4.45 (SD:1.69), 58% reported moderate physical limitations, and 14% had severe physical limitations. The median scores for perceived neighborhood safety were 2.63 (SD:0.51) and 3.93 (SD:0.41) for the groups with below median and above median safety, respectively. For neighborhood satisfaction, 27% of participants reported being dissatisfied, very dissatisfied or feeling neutral, and 73% reported being satisfied or very satisfied. The median scores for neighborhood socioeconomic deprivation were 0.74 (SD: 0.35) at the below median level and 1.43 (SD: 0.27) at the above median level. The median scores for residential instability were −0.02 (SD: 0.25) at the above median level and 0.51 (SD: 0.35) at the below median level.

Table 2 presents the unadjusted and adjusted associations of person-level factors and neighborhood factors on loneliness. After adjusting for covariates, middle-age (β: 0.36, 95% CI: 0.03,0.68) and older individuals (β: 0.44, 95% CI: 0.09,0.79) reported higher levels of loneliness compared to those aged 31 to 49. Severe physical limitations was significantly related to higher levels of loneliness in the adjusted model (β: 0.45, 95% CI: 0.08,0.82) compared to no limitations. Higher neighborhood satisfaction was associated with lower loneliness in unadjusted models only. Neighborhood socioeconomic deprivation and residential instability were not associated with loneliness in unadjusted and adjusted models. Objective neighborhood indicators showed small negative associations with loneliness. For example, the association for residential instability and loneliness (β = −0.04, 95% CI: 0.26, 0.17) suggests a potential inverse relationship. Similarly, though weaker, patterns were observed for neighborhood deprivation (β = −0.17, 95% CI: 0.39, 0.05).

Results of effect modifications by neighborhood factors on the relationship between each person-level factor and loneliness are shown in Supplementary Table 1 through 3. Table 3 presents the associations between age, physical function, and gender with loneliness, stratified by neighborhood factors that had significant interaction terms. There were significant interactions of age with both perceived neighborhood safety (β = 0.64, 95% CI: 1.28,−0.01, *p* for interaction = 0.046 for 50–65 years; β = 0.83, 95% CI: 1.43,−0.22, *p* for interaction = 0.008 for ≥65 years compared to 31–49 years) and neighborhood satisfaction (β = 0.86, 95% CI: 1.50,−0.22, *p* for interaction = 0.008 for ≥65 years compared to 31–49 years) in relation to loneliness. Among individuals who perceived their neighborhood safety as low (i.e., below median), both middle-aged adults (50–65 years) and older adults (≥65 years) reported significantly higher loneliness compared to younger adults (31–49 years), with β = 0.54 (95% CI: 0.10, 0.99) and β = 0.75 (95% CI: 0.28, 1.22), respectively. However, when perceived neighborhood safety was high (i.e., above median), these age-related differences in loneliness were not observed (*p* > 0.90 for both age groups). Stratification by neighborhood satisfaction showed that among those who were very dissatisfied/dissatisfied/or neutral, only older adults experienced significantly greater loneliness than younger adults (β = 1.01, 95% CI: 0.29, 1.73, for age ≥65 compared to ages 31–49), while no age group differences were found in the satisfied/very satisfied stratum.

Similarly, there were significant interactions between physical function and perceived neighborhood safety (β = −0.64, 95% CI: 1.13,−0.16, *p* for interaction = 0.009 for moderate limitations compared to no limitations) and neighborhood satisfaction (β = −0.82, 95% CI: 1.59, −0.06, *p* for interaction = 0.035 for severe compared to no limitations) in relation to loneliness. Stratified results showed that individuals with moderate or severe physical limitations reported higher levels of loneliness when they also perceived low neighborhood safety (β = 0.56, 95% CI: 0.19, 0.93 for moderate limitations; β = 0.54, 95% CI: 0.04, 1.05 for those with severe limitations compared to no limitations). Similarly, though weaker, patterns were observed for neighborhood satisfaction on loneliness (β = 0.77, 95% CI: 0.01, 1.56 for severe limitations and dissatisfied neighborhood).

Neither the association between age and loneliness or physical function and loneliness was moderated by neighborhood socioeconomic deprivation or residential instability. The association between gender and loneliness was only moderated by high neighborhood socioeconomic deprivation (β = 0.52, 95% CI: 0.03,1.02, *p* for interaction = 0.038 for woman compared to man). Stratified analysis demonstrated that woman reported higher levels of loneliness when they also have high neighborhood deprivation (β = 0.43, 95% CI: 0.06,0.08).

## 4. Discussion

This cross-sectional study considered multiple dimensions of neighborhood context and individual characteristics to capture the complex, multilevel nature of loneliness, guided by ETA. Age, gender, and physical limitations were considered as individual correlates to loneliness. Subjective and objective neighborhood factors were both considered in order to distinguish between individual’s perception of their environment and more objective features of their environment, interacting with individual factors to influence loneliness among adults living in predominately Black neighborhoods in Pittsburgh. Our study demonstrated that there were significant moderating effects of perceived neighborhood safety and satisfaction on the relationship between age and loneliness. Physical limitations were also linked to greater loneliness among those with low perceived safety or neighborhood satisfaction. Women had more loneliness when they also had high neighborhood socioeconomic deprivation. These findings underscore the importance of neighborhood contexts in influencing loneliness, particularly among older adults, individuals with physical limitations and women. The results contribute to understanding disparities in social well-being by illustrating that even within racially homogenous contexts, variation in one’s perception of neighborhood safety or satisfaction and neighborhood-level socioeconomic contexts may shape the experiences of loneliness. Rather than attributing disparities in loneliness solely to individual factors, our findings indicate that neighborhood environments may amplify or buffer loneliness among minoritized populations.

The independent associations of individual factors, neighborhood perceptions and objective measures, and loneliness have previously been reported in multiple studies (Feng and Astell-Burt, 2022; Hawkley and Cacioppo, 2010; Stephens and Phillips, 2022). Yet, less attention has been paid to the interactions of neighborhood factors, both subjectively and objectively, with individual factors on loneliness in adults residing in historically marginalized neighborhoods. Our results show that age-related differences in loneliness were moderated by both perceived neighborhood safety and satisfaction. Reporting higher levels of perceived neighborhood safety significantly buffered the association between older age and loneliness, suggesting that older adults may benefit more from environments where they feel secure and supported. Similarly, satisfaction with the neighborhood significantly reduced the relation of older age with loneliness. Our stratified analyses further showed that while older adults in less safe or less satisfying neighborhoods experienced higher loneliness, this age-related disparity disappeared in more subjectively positive neighborhoods. These findings are consistent with ETA, which posits that well-being in later life is shaped by the fit between person-level resources and environmental demands.

High perceived neighborhood safety and satisfaction may foster a sense of autonomy, social participation, and emotional security, counterbalancing vulnerabilities associated with aging (Stephens and Phillips, 2022; Wahl et al., 2012). Corroborating our findings, a study by Stephens and Phillips (2022) involving older adults in New Zealand found that higher housing satisfaction and neighborhood security perceptions were associated with reduced emotional loneliness. That study also demonstrated that neighborhood factors like housing satisfaction, accessibility, and social cohesion were inversely related to social loneliness, with these relationships mediated by the types of social networks older adults maintained. Together, findings from ours and others underscore the significance of perceptions about neighborhood environments in buffering against the negative effects of aging on loneliness.

Similarly, the association between physical function and loneliness was moderated by subjective neighborhood safety and satisfaction. The relationship between moderate physical limitations and loneliness was significant only among those perceiving their neighborhood as less safe. Severe limitations were also related to loneliness primarily in less satisfying neighborhoods. These findings suggest that positive neighborhood perceptions may buffer the psychosocial consequences of disability, potentially by reducing perceived barriers to social engagement or fostering supportive interpersonal interactions (Meehan et al., 2023). Feeling safe or satisfied with one’s surroundings may help offset the social withdrawal often associated with physical impairments.

In our analyses, gender was operationalized as man or woman based on the structure of the available data. Within this binary framework, we found no overall association between gender and loneliness, a pattern consistent with meta-analytic evidence indicating that the mean gender differences in loneliness are generally small and context dependent (Maes et al., 2019). However, we observed a significant moderating effect of neighborhood socioeconomic deprivation on this relationship. Stratified models further showed that in more socioeconomically deprived neighborhoods, women reported significantly higher levels of loneliness than men, whereas no gender differences were observed in less deprived neighborhoods. Prior research suggests that women often rely more heavily on proximate, community-based social networks (Pinquart and Sörensen, 2003). Women living in deprived neighborhoods may experience greater cumulative socioeconomic burden and constraints with societal roles, which can heighten vulnerability to social disconnection in this context. These findings, although cross-sectional and not causal, highlight the importance of jointly considering gendered social processes and neighborhood conditions when examining loneliness.

Objective neighborhood measures did not moderate associations between most person-level factors and loneliness, with the exception of gender. Importantly, these objective measures were derived using a lifecourse approach that captures cumulative neighborhood exposure across adulthood rather than contemporaneous conditions alone. As such, the lack of effect modification by objective neighborhood characteristics may reflect shared long-term structural environments among participants, many of whom experienced similar socioeconomic and residential contexts over time. Notably, even within these broadly comparable objective conditions, subjective neighborhood characteristics demonstrated consistent effect modification, underscoring the central role of how individuals interpret and internalize their environments. Together, these findings suggest that while objective neighborhood conditions provide important context, perceptions of neighborhood context may be more proximally linked to social experiences and feelings of loneliness.

This study has several strengths. First, we focused on a predominately Black sample, almost half of whom were 65 years or older, who reside in two historically disinvested neighborhoods with similar socioeconomic backgrounds to explore how neighborhood characteristics may shape loneliness within a population that has been historically marginalized and structurally disadvantaged. This focus enhances the relevance of our findings for addressing racial and geographic disparities in social well-being. Second, our focus on assessing both subjective and objective neighborhood measures and their moderating roles offer valuable implications for person-centered intervention that are particularly relevant in community-based settings where environmental perceptions play a key role in shaping social experiences. Third, our study also highlights the vulnerability of older adults and those with physical impairments. Our results underscore the importance of tailored strategies that address the specific needs and experiences of these subgroups. Further, our results highlight the need for interventions and policies that go beyond improving structural conditions and to also address how people feel about their neighborhood environments. Enhancing perceptions of safety, increasing satisfaction with local environments, and fostering social cohesion and belonging may be especially important for reducing loneliness among older adults and individuals with functional limitations. These person- and place-based strategies are critical for promoting health equity and social well-being in underserved communities.

Despite the strengths, there are important limitations to consider when interpreting the findings. First, the sample was restricted to adults aged 35 years and older from two predominantly Black neighborhoods in Pittsburgh, which limits generalizability to younger adults and to populations in other racial, ethnic, or geographic contexts. Because younger adults are often identified as more at risk for loneliness, our findings should be interpreted as reflecting patterns within mid-to-later adulthood among predominantly Black communities. Future research should include broader age ranges and more diverse settings to assess whether similar associations hold across the life course and in other sociodemographic groups living in neighborhoods with greater access to resources or to other racial and ethnic groups. Second, the objective neighborhood measures used in this study primarily reflect socioeconomic status and do not directly capture the availability, quality, or usability of local resources and infrastructure that may facilitate social interactions. Furthermore, these measures reflect cumulative neighborhood exposure across adulthood rather than exclusively representing current residential context. Accordingly, findings should be interpreted as associations with accumulated neighborhood environments rather than current neighborhood characteristics. Future research is needed to disentangle the relative contributions of recent versus distal neighborhood exposures and to identify potential sensitive periods. More comprehensive assessments of neighborhood context—including infrastructure quality, maintenance, walkability, access to green space, population density, housing type, and exposure to environmental stressors such as crime, inclement weather, and air pollution—may further clarify how built and social environments shape loneliness. Finally, individual and sociocultural factors likely influence how neighborhood conditions are perceived and internalized, potentially leading to differential effects on loneliness even under similar environmental conditions. Third, because participants reside in two socioeconomically similar neighborhoods, it was difficult to determine the potential impact of a full range of socioeconomic context. Future research should examine neighborhoods across a broader socioeconomic spectrum to better understand how deprived versus not deprived affect loneliness. Fourth, we recognize that our binary operationalization of gender does not capture the full spectrum of gender diversity. Future studies should adopt more inclusive measures that move beyond simplistic man/woman categories to encompass nonbinary, trans-gender, and intersex identities, as recommended by emerging scholarship (Barreto et al., 2025). Incorporating these dimensions is essential for advancing intersectional and gender-expansive understandings of loneliness. Finally, and most importantly, our findings are based on cross-sectional data, which limits causal inference. It is possible that individuals experiencing greater loneliness perceive their neighborhoods as less safe or satisfying, rather than the other way around that we presented. Longitudinal studies are needed to clarify the temporal ordering and causal relationships between neighborhood context and loneliness.

Taken together, our findings highlight the central role of neighborhood perceptions in shaping loneliness among adults living in predominantly Black and socioeconomically deprived areas. Perceptions of neighborhood satisfaction and safety appeared more salient than structural indicators in buffering the effects of aging and physical limitations on loneliness. Although gender alone was not associated with loneliness, women residing in highly deprived neighborhoods reported higher loneliness than men, suggesting that structural disadvantage may amplify gendered vulnerabilities to loneliness. These patterns underscore the need to consider both individual experiences and contextual factors when examining loneliness. Future research should extend this work using longitudinal designs across diverse neighborhood contexts and further explore how perceptions of neighborhood environments develop and influence loneliness and social well-being over time.

## Supplementary Material

supplementarytables

## Figures and Tables

**Table 1:** Sample characteristics.

	Think PHRESH Full Sample (N = 814)	Analytic Sample (N = 694)
	Mean (SD)/% (n)	Mean (SD)/% (n)
Loneliness (range: 3–9)	4.48 (1.70)	4.45 (1.69)
Perceived neighborhood safety		
Below median (range: 1–3.25)	2.63 (0.50)	2.63 (0.51)
Above median (range: 3.25–5)	3.92 (0.40)	3.93 (0.41)
Neighborhood satisfaction		
Dissatisfied/Very dissatisfied or Neutral	27% (221)	27% (187)
Satisfied/Very satisfied	73% (593)	73% (507)
Neighborhood socioeconomic deprivation		
Below median (range: 0.87–1.13)	0.75 (0.36)	0.74 (0.35)
Above median (range: 1.13–2.18)	1.43 (0.27)	1.43 (0.27)
Neighborhood residential instability		
Below median (range: 1.35–0.21)	−0.03 (0.25)	−0.02 (0.25)
Above median (range: 0.21–1.63)	0.52 (0.36)	0.51 (0.35)
Age	62.95 (12.78)	63.06 (12.61)
Age groups		
Age 31–49	17% (136)	16% (113)
Age 50–65	35% (287)	35% (245)
Age ≥65	48% (391)	48% (336)
Gender		
Woman	77% (626)	76% (524)
Man	23% (187)	25% (170)
Physical function		
No limitations	28% (227)	28% (197)
Moderate limitations	57% (465)	58% (400)
Severe limitations	14% (117)	14% (97)
Self-reported race		
Black/African American	95% (774)	95% (663)
Other	5% (37)	5% (32)
Marital status		
Married or living with a partner	11% (89)	11% (78)
Widowed	18% (144)	18% (125)
Divorced or Separated	23% (188)	22% (152)
Never married	48% (393)	49% (339)
Education level		
Less than high school	14% (116)	13% (92)
High school graduate	51% (418)	52% (359)
1–3 years of college	24% (195)	24% (169)
College degree and more	10% (83)	11% (74)
Household income (per $1K)	23.46 (19.39)	23.76 (19.52)
Neighborhood		
Hill District	58% (474)	60% (415)
Homewood	30% (245)	29% (200)
Other	12% (95)	11% (79)
Social support (range: 0–12)	9.06 (2.52)	9.12 (2.49)
General psychological distress (range: 0–24)	3.80 (4.7)	3.71 (3.99)

**Table 2:** The associations between person-level factors, neighborhood factors, and loneliness (N = 694).

Predictor (ref)	Unadjusted	Adjusted
**Person-level factors**		
Age 50–65 (31–49)	0.09 (−0.28, 0.47)	0.36 (0.03, 0.68)*
Age ≥65 (31–49)	−0.14 (−0.50, 0.23)	0.44 (0.09, 0.79)*
Woman (Man)	0.28 (−0.01, 0.57)	0.18 (−0.07, 0.43)
Moderately limited function (None)	0.69 (0.40, 0.98)***	0.25 (−0.02, 0.51)
Severely limited function (None)	1.13 (0.71, 1.54)***	0.45 (0.08, 0.82)*
**Subjective Neighborhood Factors**		
Above median neighborhood safety (below median)	−0.20 (−0.45, 0.05)	0.20 (−0.02, 0.43)
Satisfied/Very satisfied neighborhood (dissatisfied/Neutral)	−0.55 (−0.83, −0.27)***	−0.23 (−0.48, 0.03)
**Objective Neighborhood Factors**		
Above median socioeconomic deprivation (below median)	0.02 (−0.23, 0.27)	−0.04 (−0.26, 0.17)
Above median residential instability (below median)	−0.16 (−0.41, 0.09)	−0.17 (−0.39, 0.05)

Values are β coefficient (95% Confidence Interval [CI]).

* p < 0.05,

** p < 0.01,

** p < 0.001.

All models controlled for race, marital status, education level, household income, neighborhood of residence, social support, and general psychological distress. Each person-level model additionally adjusted for other person-level factors; each neighborhood model additionally adjusted for all person-level factors as well as other neighborhood measures.

**Table 3:** Stratified associations between person-level factors and loneliness by neighborhood context (N = 694).

	Neighborhood Safety	
Predictor (ref)	Below Median	Above Median
**Age 50–65 (31–49)**	0.54 (0.10, 0.99)*	−0.02 (−0.53, 0.48)
**Age ≥65 (31–49)**	0.75 (0.28, 1.22)**	0.06 (−0.48, 0.60)
**Moderately limited function (None)**	0.56 (0.19, 0.93)**	−0.04 (−0.41, 0.33)
**Severely limited function (None)**	0.54 (0.04, 1.05)*	0.40 (−0.16, 0.97)
	Neighborhood Satisfaction	
Predictor (ref)	Dissatisfied/Neutral	Satisfied
**Age 50–65 (31–49)**	0.66 (0.00, 1.33)	0.12 (−0.28, 0.51)
**Age ≥65 (31–49)**	1.01 (0.29, 1.73)**	0.17 (−0.24, 0.58)
**Moderately limited function (None)**	0.37 (−0.21, 0.95)	0.18 (−0.11, 0.46)
**Severely limited function (None)**	0.77 (−0.01, 1.56)	0.25 (−0.17, 0.67)
	Neighborhood Socioeconomic Deprivation	
Predictor (ref)	Below Median	Above Median
**Woman (Man)**	−0.05 (−0.41, 0.30)	0.43 (0.06, 0.80)*

Values are β coefficient (95% Confidence Interval [CI]).

* p < 0.05,

** p < 0.01,

** p < 0.001.

Results shown only for statistically significant interaction terms. All models controlled for race, marital status, education level, household income, neighborhood of residence, social support, and general psychological distress. Age models additionally adjusted for physical function; physical function models additionally adjusted for age. Neighborhood-specific models further adjusted for other neighborhood measures not used for stratification.

## Data Availability

Data will be made available on request.

## Appendix A. Supplementary data

Supplementary data to this article can be found online at https://doi.org/10.1016/j.healthplace.2026.103646.
