# Supplementary material for: Beyond individual correlates: The moderating roles of neighborhood factors on loneliness among adults in predominately Black neighborhoods in Pittsburgh
Source: Health Place. Author manuscript; Available in PMC 2026 Apr 15. (PMC13078169; doi:10.1016/j.healthplace.2026.103646)
Supplement: supplementarytables [file NIHMS2162523-supplement-supplementarytables.docx]

**Supplementary Table** 1. Effect Modification by Neighborhood Subjective and Objective Measures on the Association Between Age and Loneliness (N=694)

| ***Neighborhood Subjective Measures*** | | | |
| --- | --- | --- | --- |
| **Perceived Neighborhood Safety ^a^** | | **Neighborhood Satisfaction ^b^** | |
|  | β (95% CI) |  | β (95% CI) |
| Age groups (ref: Age 31-49)  Age 50-65  Age ≥65 | 0.63 (0.20, 1.06)**  0.85 (0.41, 1.28)*** | Age groups (ref: Age 31-49)  Age 50-65  Age ≥65 | 0.73 (0.17, 1.29)*  1.06 (0.51, 1.60)*** |
| Perceived neighborhood safety  (ref: below median)  Above median | 0.83 (0.30, 1.37)** | Neighborhood satisfaction  (ref: dissatisfied/very dissatisfied or Neutral)  Satisfied/Very satisfied | 0.37 (-0.17, 0.91) |
| Interaction between perceived neighborhood safety and age  Age 50-65 x Above median  Age ≥65 x Above median | -0.64 (-1.28, -0.01)*  -0.83 (-1.43, -0.22)** | Interaction between perceived neighborhood satisfaction and age  Age 50-65 x Satisfied/Very satisfied  Age ≥65 x Satisfied/Very satisfied | -0.59 (-1.27, 0.08)  -0.86 (-1.50, -0.22)** |
| ***Neighborhood Objective Measures*** | | | |
| **Socioeconomic Deprivation ^c^** | | **Residential Instability ^c^** | |
|  | β (95% CI) |  | β (95% CI) |
| Age groups (ref: Age 31-49)  Age 50-65  Age ≥65 | 0.44 (-0.01, 0.88)  0.43 (-0.03, 0.90) | Age groups (ref: Age 31-49)  Age 50-65  Age ≥65 | 0.50 (0.07, 0.93)*  0.49 (0.03, 0.95)* |
| Socioeconomic deprivation  (ref: below median)  Above median | 0.02 (-0.51, 0.54) | Residential instability  (ref: below median)  Above median | -0.01 (-0.53, 0.52) |
| Interaction between socioeconomic deprivation and age  Age 50-65 x Above median  Age ≥65 x Above median | -0.18 (-0.81, 0.46)  0.01 (-0.60, 0.61) | Interaction between residential instability and age  Age 50-65 x Above median  Age ≥65 x Above median | -0.37 (-1.01, 0.26)  -0.08 (-0.68, 0.53) |

^a^ adjusted for gender, physical function, race, marital status, education level, household income, neighborhood of residence, social support, general psychological distress, neighborhood satisfaction, neighborhood socioeconomic deprivation, and residential instability.

^b^ adjusted for gender, physical function, race, marital status, education level, household income, neighborhood of residence, social support, general psychological distress, neighborhood perceived safety, neighborhood socioeconomic deprivation, and residential instability.

^c^ adjusted for gender, physical function, race, marital status, education level, household income, neighborhood of residence, social support, and general psychological distress.

*p<0.05, **p<0.01, **p<0.001.

**Supplementary Table** 2. The Effect Modification by Neighborhood Subjective and Objective Measures on the Association Between Gender and Loneliness (N=694)

| ***Neighborhood Subjective Measures*** | | | |
| --- | --- | --- | --- |
| **Perceived Neighborhood Safety ^a^** | | **Neighborhood Satisfaction ^b^** | |
|  | β (95% CI) |  | β (95% CI) |
| Gender (ref: Man)  Woman | 0.19 (-0.17, 0.55) | Gender (ref: Man)  Woman | 0.42 (-0.07, 0.92) |
| Perceived neighborhood safety  (ref: below median)  Above median | 0.04 (-0.46, 0.54) | Neighborhood satisfaction  (ref: dissatisfied/very dissatisfied or Neutral)  Satisfied/Very satisfied | -0.01 (-0.51, 0.49) |
| Interaction between perceived neighborhood safety and gender  Woman x Above median | 0.04 (-0.46, 0.54) | Interaction between perceived neighborhood satisfaction and gender  Woman x Satisfied/Very satisfied | -0.28 (-0.85, 0.28) |
| ***Neighborhood Objective Measures*** | | | |
| **Socioeconomic Deprivation ^c^** | | **Residential Instability ^c^** | |
|  | β (95% CI) |  | β (95% CI) |
| Gender (ref: Man)  Woman | -0.06 (-0.40, 0.28) | Gender (ref: Man)  Woman | 0.31 (-0.03, 0.64) |
| Socioeconomic deprivation  (ref: below median)  Above median | -0.44 (-0.88, -0.01)* | Residential instability  (ref: below median)  Above median | 0.02 (-0.41, 0.45) |
| Interaction between socioeconomic deprivation and gender  Woman x Above median | 0.52 (0.03, 1.02)* | Interaction between residential instability and gender  Woman x Above median | -0.26 (-0.75, 0.24) |

^a^ adjusted for age, physical function, race, marital status, education level, household income, neighborhood of residence, social support, general psychological distress, neighborhood satisfaction, neighborhood socioeconomic deprivation, and residential instability.

^b^ adjusted for age, physical function, race, marital status, education level, household income, neighborhood of residence, social support, general psychological distress, neighborhood perceived safety, neighborhood socioeconomic deprivation, and residential instability.

^c^ adjusted for age, physical function, race, marital status, education level, household income, neighborhood of residence, social support, and general psychological distress.

*p<0.05, **p<0.01, **p<0.001.

**Supplementary Table** 3. The Effect Modification by Neighborhood Subjective and Objective Measures on the Association Between Physical Limitation and Loneliness (N=694)

| ***Neighborhood Subjective Measures*** | | | |
| --- | --- | --- | --- |
| **Perceived Neighborhood Safety ^a^** | | **Neighborhood Satisfaction ^b^** | |
|  | β (95% CI) |  | β (95% CI) |
| Physical function (ref: No limitations)  Moderate limitations  Severe limitations | 0.57 (0.22, 0.92)**  0.57 (0.09, 1.05)* | Physical function (ref: No limitations)  Moderate limitations  Severe limitations | 0.51 (0.03, 1.00)*  1.05 (0.38, 1.71)** |
| Perceived neighborhood safety  (ref: below median)  Above median | 0.60 (0.19, 1.00)** | Neighborhood satisfaction  (ref: dissatisfied/very dissatisfied or Neutral)  Satisfied/Very satisfied | 0.10 (-0.36, 0.56) |
| Interaction between perceived neighborhood safety and physical function  Moderate limitations x Above median  Severe limitations x Above median | -0.64 (-1.13, -0.16)**  -0.16 (-0.86, 0.54) | Interaction between perceived neighborhood satisfaction and physical function  Moderate limitations x Satisfied/Very satisfied  Severe limitations x Satisfied/Very satisfied | -0.35 (-0.90, 0.20)  -0.82 (-1.59, -0.06)* |
| ***Neighborhood Objective Measures*** | | | |
| **Socioeconomic Deprivation ^c^** | | **Residential Instability ^c^** | |
|  | β (95% CI) |  | β (95% CI) |
| Physical function (ref: No limitations)  Moderate limitations  Severe limitations | 0.18 (-0.17, 0.53)  0.36 (-0.15, 0.86) | Physical function (ref: No limitations)  Moderate limitations  Severe limitations | 0.26 (-0.10, 0.61)  0.20 (-0.32, 0.72) |
| Socioeconomic deprivation  (ref: below median)  Above median | -0.16 (-0.56, 0.24) | Residential instability  (ref: below median)  Above median | -0.23 (-0.63, 0.17) |
| Interaction between socioeconomic deprivation and physical function  Moderate limitations x Above median  Severe limitations x Above median | 0.15 (-0.33, 0.64)  0.18 (-0.51, 0.97) | Interaction between residential instability and physical function  Moderate limitations x Above median  Severe limitations x Above median | -0.02 (-0.50, 0.46)  0.46 (-0.23, 1.14) |

^a^ adjusted for age, gender, race, marital status, education level, household income, neighborhood of residence, social support, general psychological distress, neighborhood satisfaction, neighborhood socioeconomic deprivation, and residential instability.

^b^ adjusted for age, gender, race, marital status, education level, household income, neighborhood of residence, social support, general psychological distress, neighborhood perceived safety, neighborhood socioeconomic deprivation, and residential instability.

^c^ adjusted for age, gender, race, marital status, education level, household income, neighborhood of residence, social support, and general psychological distress.

*p<0.05, **p<0.01, **p<0.001.

**Supplementary Table** 4. Correlations Between Subjective and Objective Neighborhood Measures (N=694)

|  | **Subjective and Objective Neighborhood Measures** | | | |
| --- | --- | --- | --- | --- |
|  | Perceived neighborhood safety | Neighborhood satisfaction | Neighborhood socioeconomic deprivation | Neighborhood residential instability |
| Perceived neighborhood safety | 1 |  |  |  |
| Neighborhood satisfaction | 0.42 | 1 |  |  |
| Neighborhood socioeconomic deprivation | -0.06 | -0.10 | 1 |  |
| Neighborhood residential instability | 0.11 | 0.02 | 0.09 | 1 |
